# Prevalence of internet use disorder and associated psychiatric comorbidities in youth patients presenting to tertiary care center

**DOI:** 10.1192/j.eurpsy.2023.1401

**Published:** 2023-07-19

**Authors:** R. Ghimire, N. Sapkota, D. R. Shakya, R. G. Joshi, S. Nepal

**Affiliations:** 1 Dhaulagiri Hospital, Baglung; 2 Psychiatry, Patan Academy of Health Sciences, Kathmandu; 3 Psychiatry, BPKIHS, Dharan, Nepal

## Abstract

**Introduction:**

Internet use has become integral part of daily living in current world; at the same time its misuse is likely to cause addiction and negative impact in mental health. Internet addiction is similar to other substance addiction and has been associated with different psychiatric comorbidities.

**Objectives:**

To study prevalence of internet use disorder in youth patients,to find the association of psychiatric comorbidities with pattern of internet use and to study socio-demographic variables.

**Methods:**

An institutional based cross-sectionalstudy was carried out in psychiatric out-patientdepartment of tertiary hospital. A total of 146 youth patients (15-24 years) who consented for the study were enrolled. Psychiatric diagnosis was made by the consultant psychiatrist using ICD-10/DSR criteria.

**Results:**

Out of total 146 patients with mean age 19.99 years, internet use was found in 89.7% of youth patients out of which 63.7% used in single device and 26.3% in multiple devices. Internet addiction was found in 67.8% youth patients and internet gaming in 33.6% patients. Higher level of internet addiction was found in adolescent (15-19 years) than adult (20-24 years) with statistically significant p value 0.014; patients from middle and higher socio-economic condition than low socio-economic conditionwith statistically significant p value 0.024; patients from uran region compared to semi-urban and rural with statistically significant p value 0.000; and patient using internet gaming with statistically significant p vallue 0.000. Among psychiatric diagnosis, ANxiety, depression and subtance use disorders were associated with internet use disorder however were statistically insignificant.

**Conclusions:**

Internet addiction is high in youth patients; adolescent (15-19 years) compared to adults (20-24 years). Anxiety, Depression and substance use disorders had higher association with internet use disorder.

**Disclosure of Interest:**

None Declared

